# Absolute quantification of cerebral metabolites using two‐dimensional proton MR spectroscopic imaging with quantitative MRI‐based water reference

**DOI:** 10.1002/mrm.70027

**Published:** 2025-08-13

**Authors:** Dennis C. Thomas, Seyma Alcicek, Andrei Manzhurtsev, Elke Hattingen, Katharina J. Wenger, Ulrich Pilatus

**Affiliations:** ^1^ Goethe University Frankfurt University Hospital, Institute of Neuroradiology Frankfurt am Main Germany; ^2^ University Cancer Center Frankfurt Frankfurt am Main Germany; ^3^ Frankfurt Cancer Institute Frankfurt am Main Germany; ^4^ German Cancer Research Center Heidelberg and German Cancer Consortium Heidelberg Germany

**Keywords:** brain tumors, quantitative MRI, spectroscopy

## Abstract

**Purpose:**

Metabolite concentrations are valuable biomarkers in brain tumors (BTs). However, absolute quantification of metabolites using MR spectroscopy requires a correction of water relaxation using time‐consuming quantitative MRI (qMRI) sequences in addition to a lengthy two‐dimensional spectroscopic water‐reference acquisition. The goal of this work was to develop and validate a fast quantification method where a two‐dimensional spectroscopic water reference is obtained using qMRI and a single‐voxel stimulated‐echo acquisition mode (STEAM) sequence.

**Methods:**

The semi‐adiabatic localization by adiabatic selective refocusing (sLASER) sequence was used for MR spectroscopy imaging (MRSI) acquisition. A single‐voxel unsuppressed water signal was acquired using a STEAM sequence. A qMRI protocol was also acquired, and the H_2_O map was calibrated based on the STEAM signal to obtain the spectroscopic water reference (proposed method). Five healthy volunteers and one BT patient were scanned at 3 T. Concentrations obtained using the proposed and two reference methods—one where water‐relaxation effects were corrected using literature values (reference method) and one where they were corrected using qMRI‐derived values (reference method with qMRI)—were compared.

**Results:**

In healthy subjects, white‐matter metabolite concentrations obtained using water relaxation using literature values (reference method) significantly differed from those using individual‐specific corrections (reference method with qMRI and proposed method). Bland–Altman analyses revealed a very low bias and standard deviation of the differences between the reference method with qMRI and the proposed method (bias < 0.5% and standard deviation < 10%). The BT regions showed an approximate 35% underestimation of metabolite concentrations using the reference method.

**Conclusion:**

For metabolite quantification, accurate water referencing with individual‐specific corrections for water relaxation times was obtained in 8 min using the proposed method.

## 1 INTRODUCTION

Proton magnetic resonance spectroscopy (^1^H‐MRS) is a powerful tool, capable of measuring in vivo metabolite concentrations.^1^ In brain tumors (BTs), changes in the relative and absolute concentrations of metabolites can serve as diagnostic and therapeutic biomarkers.^2^, ^3^, ^4^ To study the differences in the metabolite concentrations between patient and healthy cohort or between tumor tissue and healthy tissue in the same patient, the metabolite signal has to be referenced either to that of another metabolite or to that of water.^5^, ^6^ This is because the signal amplitude at a certain region depends not only on the concentration of the metabolite, but also on factors that are hard to determine, like the receiver gain (B_1_^−^), the transmitter gain (B_1_^+^), and a scanner‐dependent intensity scaling factor.^5^, ^7^ These factors could vary among regions, subjects, and scanners. In the past, concentration ratios (e.g., total N‐acetylaspartate/total creatine [tNAA/tCr]) were widely reported for clinical use. However, this approach suffers from the ambiguity of attributing the changes to the metabolite of interest (in the numerator) or the reference metabolite (in the denominator) and the fact that small or inaccurate values for the denominator produce large variance in the metabolite ratios.^8^ Another concern is the stability of the reference metabolite across age, gender, pathology, or other factors.^6^ For these reasons, it is advised that the metabolite concentrations be obtained by referencing the metabolite signal to that of water.^6^, ^7^, ^9^, ^10^, ^11^, ^12^

Semi‐quantitative MRS can be achieved by calculating the ratio of each metabolite to the unsuppressed tissue water signal from the same voxel of interest (VOI), while assuming a standard MR‐visible water content in the tissue.^13^ However, for full quantification of metabolite concentrations with the internal water reference, it is necessary to adjust both water and metabolite signals for relaxation effects, account for tissue partial‐volume effects, and correct for regional variations of the water signal (in relation to B_1_ inhomogeneity and eddy currents).^9^ Typically, the metabolite signal is acquired with a water‐suppressed MRS sequence followed by the acquisition of the same MRS sequence without water suppression. The fitted metabolite signal intensity in a voxel is then divided by the fitted water signal intensity obtained from the same voxel, and relaxation effects of water and metabolites are corrected for with relaxation times obtained from literature.^9^ However, this method of quantification has two disadvantages: First, the acquisition of both water suppressed and unsuppressed spectra under the same conditions nearly doubles acquisition time, which is undesirable for already lengthy MR spectroscopy imaging (MRSI) protocols. Second, using literature values of water relaxation may not be accurate, as the relaxation values in brain pathologies differ strikingly.^14^, ^15^ Even in healthy subjects, the water‐relaxation times show significant variations between subjects and brain regions of different tissue water density, making a single correction factor insufficient.^16^, ^17^

Because using literature water relaxation values for white matter (WM), gray matter (GM), and cerebrospinal fluid (CSF) could lead to inaccuracies in metabolite quantification, some studies have used quantitative MRI (qMRI) to their quantification approach.^18^, ^19^, ^20^, ^21^, ^22^ Notably, for MRSI, Gasparovic et al. carried out a detailed quantification approach to correct for the water‐relaxation times by measuring the whole‐brain water content (H_2_O), T_1_ and T_2_ maps, and correcting the acquired unsuppressed water signal for T_1_ and T_2_ relaxation.^18^ The H_2_O map was used for obtaining molar concentrations of the metabolites from the molal concentrations. Although the corrections proposed in this study lead to accurate metabolite quantification, the very long acquisition time for obtaining the corrected water reference (45 min) is a deterrent for routine application. The long acquisition time is partly because both the unsuppressed water spectrum and the qMRI maps are acquired. Recently, it was shown that the acquisition time for absolute quantification can be significantly decreased using a fast qMRI protocol. However, even with this approach, it becomes imperative to acquire the unsuppressed water signal. This is a redundant approach, as the spatial information encoded in the two‐dimensional (2D) MRSI unsuppressed water spectrum is also present in the qMRI maps, namely in the H_2_O and T_1_ maps.^23^, ^24^, ^25^, ^26^

In the current work, we test the feasibility of synthesizing a 2D MRSI of unsuppressed water combining qMRI techniques with a single‐voxel stimulated echo acquisition mode (STEAM) acquisition. The latter was used to calibrate the H_2_O maps to the proton (^1^H) MRSI data. We investigate whether lower scan times can be achieved using this approach without significant impact of the accuracy of metabolite quantification.

The aims of this work were:

1. To develop a fast, individual‐specific ^1^H‐MRSI quantification protocol (˜8 min) without the need of an additional unsuppressed MRSI water reference, using qMRI and a single‐voxel (SV) STEAM sequence for calibration.
2. To evaluate the differences between the obtained metabolite concentrations using the proposed method and currently recommended approaches (according to the consensus papers^1–4^,^6^, ^8^, ^27^ in 5 healthy subjects and 1 BT patient.

## 2 METHODS

Consider that we are interested in measuring the concentration of a metabolite “M” ([M]). As outlined by Ernst et al.,^28^ [M] can be expressed as molal units (mol/kg of water) or molar units (mol/L of tissue). To elaborate, when expressed in molal units, ${\left[M\right]}_{\text{molal}}$ refers to the number of moles of “M” present in 1 kg of “tissue water,” and when expressed in molar units, ${\left[M\right]}_{\text{molar}}$ refers to the number of moles of “M” present in 1 L of “tissue,” which includes the non‐water tissue and the intracellular/extracellular (IC/EC) tissue water, as follows:

(1) $${\left[M\right]}_{\text{molal}}=\frac{\mathrm{No}.\;\text{of moles of metabolite}}{\text{Mass of water}}$$

(2) $${\left[M\right]}_{\text{molar}}=\frac{\mathrm{No}.\;\text{of moles of metabolite}}{\text{Volume of tissue}}$$

Note that, in this study, [M] excludes the CSF water, as most MRS detectable metabolites are not expected to be present in the CSF. The number of moles of a metabolite in each voxel is proportional to the metabolite signal, $S_{M}$, in the spectra of the corresponding voxel of the acquired MRSI data. To obtain absolute concentrations of metabolites, $S_{M}$ needs to be calibrated, which, as mentioned in the Introduction, is usually achieved by recording the unsuppressed tissue water signal. As recommended in the 2020 consensus paper on preprocessing, analysis, and quantification in MRS,^6^ a water‐unsuppressed scan should ideally be recorded under the same conditions as the spectroscopic data. Relaxation corrections (T_1_, T_2_) for water can then be performed based on literature values (if segmented images are available)^9^ or based on the relaxation times measured using a qMRI protocol.^18^, ^19^ Because the approach with literature values is routinely used, it will be referred to as the “reference method” (Ref‐method).^9^ The approach using values measured with qMRI will be referred to as the “reference method with qMRI” (Ref‐method‐with‐qMRI).^18^ To avoid the lengthy acquisition of the spectroscopic water reference, we propose a third method that uses an unsuppressed SV STEAM sequence to calculate a spectroscopic water reference map from qMRI data. This method will be referred to as the “proposed method” (Proposed‐method). Although most of the theory behind the Ref‐method and Ref‐method‐with‐qMRI have been explained in previous publications, we provide an overview of the theory for these approaches in the Supporting Information, for sake of clarity. We describe the Proposed‐method in detail next.

### 2.1 Proposed method (“Proposed‐method”)

#### 2.1.1 Synthesis of STEAM MRSI water reference

**FIGURE 1:**
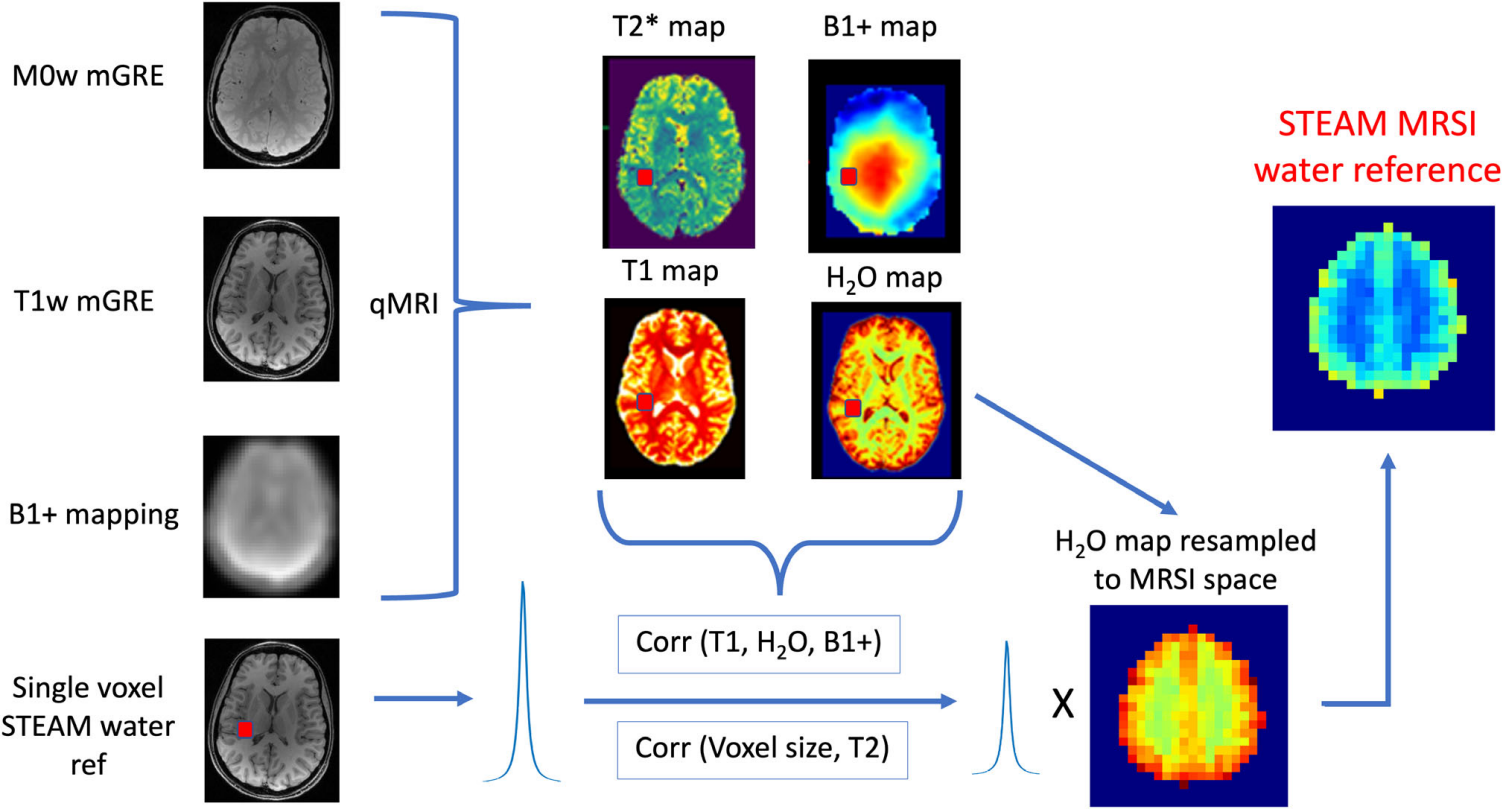
Graphical depiction of the Proposed‐method. For the quantitative MRI (qMRI) protocol, two multi‐echo gradient‐echo (mGRE) images (M0w/T1w) images and two echo‐planar images ($45^{{}^{\circ}}$/90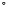) are obtained. A single‐voxel, water‐unsuppressed, stimulated echo acquisition mode (STEAM) signal is obtained from a voxel placed in the normal‐appearing white matter. The STEAM water signal is then corrected for T_1_, T_2_, B_1_^+^, and voxel size, to obtain the corrected water signal which is then used to calibrate the $H_{2}O_{\mathrm{map}}$, which yields the STEAM–MR spectroscopy imaging (MRSI) water reference ($2D_{{\text{STEAM}}_{W}}$).

The proposed method for absolute quantification of metabolites is graphically depicted in Figure 1. T_1_, T_2_*, B_1_^+^, and H_2_O maps ($T1_{\mathrm{map}},T{2^{*}}_{\mathrm{map}},B1_{\mathrm{map}}\&H_{2}O_{\mathrm{map}}$) are obtained using a vendor‐based qMRI.^29^ The $H_{2}O_{\mathrm{map}}$ was used to synthesize a spectroscopic water reference. To do this, for calibration, a water‐unsuppressed SV STEAM sequence is run with the voxel positioned in the normal‐appearing WM (NAWM) (voxel size = 10^3^ mm^3^). This measured water signal is labeled ${{\mathrm{sv}}_{\text{STEAM}}}_{W}$. The value of ${{\mathrm{sv}}_{\text{STEAM}}}_{W}$ is corrected for the B_1_^+^ inhomogeneity, T_1_ relaxation, and T_2_ relaxation using the B_1_^+^, T_1_, and T_2_* values of that voxel obtained from the qMRI maps. Because the fast qMRI protocol used does not produce a $T2_{\mathrm{map}}$, the T_2_ value of the water in the STEAM voxel is estimated using the T_2_* map. We propose a constant calibration for obtaining $T_{2_{\text{STEAM}}}$ from ${{T_{2}}^{*}}_{\text{STEAM}}$: $T_{2_{\text{STEAM}}}={{T_{2}}^{*}}_{\text{STEAM}}\cdot \left(\frac{\text{Literature}\;\mathrm{WM}\;T2\;\text{value}\;}{\text{Literature}\;\mathrm{WM}\;T2^{*}\;\text{value}\;}\right)$. LiteratureWMT2value and $\text{Literature}\;\mathrm{WM}\;T2^{*}\;\text{value}$ were set to 59 ms (Küppers et al.^33^) and 47 ms (Thomas et al.^29^), respectively. Furthermore, ${{\mathrm{SV}}_{\text{STEAM}}}_{W}$ is corrected for the difference in the voxel size between STEAM and sLASER, as follows:

(3) $${{{\mathrm{sv}}_{\text{STEAM}}}_{W}}_{\text{corr}}={{\mathrm{sv}}_{\text{STEAM}}}_{W}\cdot T1_{\text{corr}}\cdot T2_{\text{corr}}\cdot {B1}_{\text{corr}}^{+}\cdot \text{voxelcorr}$$

where

(4) $$T1_{\text{corr}}=\left(1-\exp\left(\frac{\mathrm{TE}}{2}+\mathrm{TM}-\frac{\mathrm{TR}}{T_{1_{\text{STEAM}}}}\right)\right)\cdot \exp\left(\frac{-\mathrm{TM}}{T_{1_{\text{STEAM}}}}\right)$$

(5) $$T2_{\text{corr}}=\frac{1}{e^{\frac{-\mathrm{TE}}{T_{2_{\text{STEAM}}}}}}$$

(6) $${B1}_{\text{corr}}^{+}=\frac{\sin\left(\alpha \right)}{\sin{\left(B1^{+\cdot }\alpha \right)}^{3}}$$

(7) $$\text{voxelcorr}=\frac{\text{voxelsiz}e_{\text{sLASER}}}{\text{voxelsiz}e_{\text{STEAM}}}$$

Equation (4) has been taken from Gogishvili et al.^30^ Equation (6) has been adapted from one described by Helms.^31^ The values of $T_{1_{\text{STEAM}}}$, $T_{2_{\text{STEAM}}}$, and ${{T_{2}}^{*}}_{\text{STEAM}}$ refer to the T_1_, T_2_, and T_2_* of water in the STEAM voxel; α refers to the nominal flip angle; and TM refers to the mixing time.

Finally, $s{{v_{\text{STEAM}}}_{W}}_{\text{corr}}$ is used to calibrate the $H_{2}O_{\mathrm{map}}$, extending the SV measurement to synthesize a calibrated STEAM‐MRSI water reference ($2D_{{\text{STEAM}}_{W}}$), as follows:

(8) $$2D_{{\text{STEAM}}_{W}}=\frac{{{{\mathrm{sv}}_{\text{STEAM}}}_{W}}_{\text{corr}}\cdot H_{2}O_{{\mathrm{map}}_{\text{MRSI}}}}{\sum H_{2}O_{{\mathrm{map}}_{\mathrm{HR}}}\left(i\right)}$$

where i refers to the voxels in the high‐resolution $H_{2}O_{\mathrm{map}}$ ($H_{2}O_{{\mathrm{map}}_{\mathrm{HR}}}$), which lie within the 10^3^ mm^3^ STEAM voxel, and $H_{2}O_{{\mathrm{map}}_{\text{MRSI}}}$ refers to the $H_{2}O_{\mathrm{map}}$ resliced into the MRSI space and corrected for the broader point spread function of the MRSI acquisition.

#### 2.1.2 Metabolite concentration quantification

The value of $2D_{{\text{STEAM}}_{W}}$ can be used as the spectroscopic water reference ($S_{w}$), and the absolute concentration of the metabolites can be computed as follows:

(9) $${\left[M\right]}_{\text{molal}}=\frac{S_{M}}{2D_{{\text{STEAM}}_{W}}\cdot \left(1-f_{{\mathrm{CSF}}_{H2O}}\right)\cdot R_{M}}\cdot {\left[H_{2}O\right]}_{\text{molal}}$$

(10) $$R_{M}=e^{\frac{-\mathrm{TE}}{T_{2_{M}}}}\cdot \left(1-e^{\frac{-\mathrm{TR}}{T_{1_{M}}}}\right)$$

The values of T1_M_ and T2_M_ refer to the metabolite T_1_ and T_2_ relaxation times, which are obtained from literature for GM and WM regions.^29^, ^32^, ^33^, ^34^, ^35^ The value of $R_{M}$ denotes the metabolite relaxation correction. The molality of water (${\left[H_{2}O\right]}_{\text{molal}}$) is 55.1 mmol/kg; $f_{{\mathrm{CSF}}_{H2O}}$ refers to the CSF molal water fraction, as described by Gasparovic et al.^9^ and is calculated as shown in Eq. (11); $d_{\mathrm{CSF}}$ represents the CSF water density and was assumed to be equal to unity in this study; and $f_{\mathrm{CSF}}$ denotes the CSF volume fraction obtained from segmentation of the T_1_‐weighted image.

(11) $$f_{{\mathrm{CSF}}_{H2O}}=\frac{f_{\mathrm{CSF}}\cdot d_{\mathrm{CSF}}}{H_{2}O_{\mathrm{map}}}$$

Note that in Eq. (9), the factor “$R_{H2O}$,” which denotes the correction for water relaxation, is omitted. This is because $2D_{{\text{STEAM}}_{W}}$, which is obtained using Eq. (8) is already corrected for the water‐relaxation effects. The following equation describes the relation between ${\left[M\right]}_{\text{molar}}$ and ${\left[M\right]}_{\text{molal}}$ metabolite concentrations:

(12) $${\left[M\right]}_{\text{molar}}={\left[M\right]}_{\text{molal}}\cdot \frac{\left(H_{2}O_{\mathrm{map}}-f_{\mathrm{CSF}}\right)}{\left(1-f_{\mathrm{CSF}}\right)}\cdot \rho_{w}$$

where $\rho_{w}$ represents the density of pure water [kg/L] and was assumed to be equal to unity. There are two major differences between the Ref‐method‐with‐qMRI and the Proposed‐method:

1. The T_2_ map for the Proposed‐method is not directly acquired. The T_2_ value of the STEAM voxel is estimated from the T_2_* value in the STEAM voxel using a constant correction factor (1.255).
2. The spectroscopic unsuppressed water reference is not directly acquired for the proposed method but instead calculated by acquiring a SV STEAM sequence and calibrating the H_2_O map using the STEAM signal.

### 2.2 Study protocol

#### 2.2.1 MRS

The study protocol was approved by the institutional review board (Ethics Committee, University Hospital Frankfurt, Project Number 2022‐1035), and written informed consent was obtained from all subjects and patients. In vivo measurements were performed on 5 healthy subjects and 1 BT patient on a whole‐body 3T MR Scanner (MAGNETOM Prisma [VE11C]; Siemens Healthineers, Erlangen, Germany). An additional BT patient was scanned to demonstrate the effect of STEAM voxel placement on the quantification. The MRI sequence protocol was the same for the healthy subjects and the BT patients. MRSI was acquired with a sLASER MRSI sequence^36^ with acquisition parameters as detailed in Table 1. For the “Ref‐method” and “Ref‐method‐with‐qMRI,” the spectroscopic water reference was acquired with the same parameters as the sLASER MRSI but without water suppression. For the Proposed‐method, a 10^3^‐mm^3^ SV STEAM sequence was acquired in the NAWM, with no water suppression.

**TABLE 1:** Acquisition parameters of the spectroscopic sequences.

	sLASER MRSI	sLASER MRSI water reference	SV STEAM
TR	2000 ms	2000 ms	10 000 ms
TE	40 ms	40 ms	20 ms
TM	–	–	10 ms
FlFA	90	90	90
BW	2000 Hz	2000 Hz	1200 Hz
Interpolated resolution	7.5 × 7.5 × 12 mm^3^	7.5 × 7.5 × 12 mm^3^	10 × 10 × 10 mm^3^
Saturation bands	Yes (as shown in Figure S2C)	Yes (as shown in Figure S2C)	–
Matrix size	20 × 20	20 × 20	–
Averages	2 (weighted phase encoding)^51^	1 (circular phase encoding)	1
In‐plane FOV	240 × 240 mm^2^	240 × 240 mm^2^	–
No. of slices	1	1	–
Slice thickness	12	12	–
Water suppression	CHESS	–	–
TA	10:52 min	8 min	10 s

Abbreviations: BW, bandwidth; FA, flip angle; FOV, field of view; MRSI, MR spectroscopic imaging; SV, single voxel; STEAM, stimulated‐echo acquisition mode; TE, echo time; TA, acquisition time; TM, mixing time; TR, pulse repetition time.

For the 5 healthy subjects, the spectroscopic slice was positioned above the corpus callosum, so that no part of the lateral ventricles was within the VOI. For the BT patients, the VOI was positioned to cover the solid part of the tumor and the contralateral healthy tissue. Outer‐volume suppression bands (saturation bands) were used to suppress unwanted signals from outside the VOI in all volunteers and patients (as shown in Figure S2C).

To compare the concentrations obtained using MRSI STEAM water reference and MRSI sLASER water reference, in one other BT patient, the following were acquired: water‐suppressed MRSI sLASER (TE = 40 ms), water‐unsuppressed MRSI sLASER (TE = 40 ms), and water‐unsuppressed MRSI STEAM (TE = 40 ms). The metabolite concentrations obtained using the two water reference acquisitions were obtained and compared.

#### 2.2.2 qMRI

For the Ref‐method‐with‐qMRI and Proposed‐method, an 8‐min vendor‐based protocol for obtaining whole‐brain T_1_, H_2_O, T_2_*, and quantitative susceptibility maps was acquired with the protocol described by Thomas et al.^29^ This included measuring two multi‐echo gradient‐echo (mGRE) images (M0w and T1w) and two echo‐planar images for B_1_ mapping (Figure 1). Additionally, for the Ref‐method‐with‐qMRI, T_2_ mapping with three single‐echo spin‐echo images and 20 slices (to reduce acquisition time) was acquired. The slices were positioned to cover the VOI of the MRSI slab. Acquisition parameters are detailed in Table 2.

**TABLE 2:** Acquisition parameters of quantitative MRI (qMRI) sequences.

	mp‐qMRI protocol for both Ref‐method‐ with‐qMRI and Proposed‐method		Additional T_2_ mapping for the Ref‐method‐with‐qMRI
	M0w/ T1w	EPI 45°/90°	
TR	30 ms	10 000 ms	10 000 ms
TE0	3.2 ms	21 ms	25 ms
∆TE	4.5 ms	–	25 ms
nTE	6	1	3
FA	5/32	45/90	90
BW	350 Hz/Px	2298 Hz/Px	600 Hz/Px
Resolution	1.2 × 1.6 × 1.2 mm^3^ (interp: (1.2)^3^ mm^3^)	4.0 × 4.0 × 2.0 mm^3^	1.5 × 1.5 × 1.5 mm^3^
Matrix size	192 × 144	64 × 64	
Averages	1	1	1
FOV	224 × 217 mm	256 × 256 mm	224 × 224
No. of slices	1	60	20
Slice thickness	1.2 mm	2.0 mm	1.5 mm
Parallel imaging	GRAPPA, acc. = 2	–	
Slice partial Fourier	6/8	–	
Phase partial Fourier	6/8	–	
Total TA	7 min	1 min	6:20 min

Abbreviations: BW, bandwidth; EPI, echo‐planar imaging; FA, flip angle; FOV, field of view; TA, acquisition time; TE, echo time; TR, pulse repetition time.

### 2.3 Data processing

#### 2.3.1 Metabolite spectra fitting

Spectroscopic data were processed using the LCModel software.^37^ Metabolite basis sets were simulated for the sLASER pulse sequence at echo time (TE) = 40 ms using the jMRUI plug‐in NMR‐ScopeB (version 6.0, available at http://www.jmrui.eu ^38^) with prior knowledge of chemical shifts and J‐coupling. In the control file used for LCModel processing, the parameters “atth2o,” “attmet,” and “wconc,” which were responsible, respectively, for correction of the water‐relaxation effects, metabolite relaxation effects, and water density, were set equal to 1, avoiding correction of water and metabolite relaxation effects and water density. The reslicing and averaging of anatomical and quantitative data into the spectroscopic domain and all the quantification steps were carried out in *Python* v3.9.12, using home‐written scripts. The open‐source *Python* packages used for the postprocessing and analysis are Numpy, Pandas, and Nibabel.

#### 2.3.2 Quantification of metabolite concentrations

T_1_, H_2_O, T_2_*, and quantitative susceptibility mapping was carried out with the postprocessing steps detailed by Thomas et al.^29^ For T_2_ mapping, the three spin‐echo images acquired at TEs of 25, 50, and 75 ms were voxel‐wise‐fitted using the following equation:

(19) $$S\left[\mathrm{TE}\right]=e^{\frac{-\mathrm{TE}}{T_{2}}}$$

where S[TE] denotes the signal at TE.

For the Ref‐method, the unsuppressed sLASER water reference scan was given as the water reference. LCModel computes the concentration of the metabolites by normalizing the metabolite signal to this water signal. The LCModel output corresponds to $\frac{S_{\mathrm{met}}}{S_{w}}$. Then, the corrections for water relaxation, metabolite relaxation, and CSF partial volume effect were carried out as detailed in the Supporting Information. The literature values used for the water and metabolite relaxation correction are given in Tables S1 and S2. The first‐echo image of the T_1_‐weighted mGRE was used for the WM, GM, and CSF segmentation using *SPM*. For the Ref‐method‐with‐qMRI, the corrections were carried out as detailed in the Supporting Information. Instead of using values from literature for T_1_, T_2_, and water content (H_2_O) of WM, GM, and CSF, the actual measured T_1_, T_2_, and H_2_O maps were used. CSF segmentation was performed for correction of CSF partial volume effect.

For the Proposed‐Method, the 2D spectroscopic water reference was obtained using qMRI and STEAM acquisition as detailed in the Methods. The STEAM unsuppressed water signal was propagated to form a STEAM‐MRSI array of the same matrix size as that of the sLASER metabolite acquisition and given as the water reference in LCModel. The metabolite concentrations that were obtained from LCModel were then corrected using Eqs. ((3), (4), (5), (6), (7), (8), (9), (10), (11))–((3), (4), (5), (6), (7), (8), (9), (10), (11)).

For the BT patient data set, the quantification of the metabolite concentrations differed from the healthy volunteers in two ways: First, because no T_2_ map was acquired due to acquisition time constraints, the T_2_ map was estimated from the T_2_* map by multiplying it by $\left(\frac{\text{Literature}\;\mathrm{WM}\;T2\;\text{value}\;}{\text{Literature}\;\mathrm{WM}\;T2^{*}\;\text{value}\;}\right)$, which as described before, was set to $\left(\frac{59}{47}\right)$.^29^, ^33^ Second, for the tumor patient, it happened to be the case that the STEAM voxel was positioned away from the MRSI slice in the through‐slice direction. In this case, to account for the inaccuracies in the “Prescan normalize” correction of receiver inhomogeneity, an additional correction was carried out. The respective calibration factor K, was obtained by dividing the signal from a WM voxel (WM > 98%) of the MRSI water reference (corrected for voxel size and relaxation) by the single‐voxel STEAM water signal (corrected for voxel size and relaxation). The final metabolite concentration maps obtained using the ‘Proposed‐method’ were multiplied by “K.” To demonstrate that the use of K is unnecessary if the STEAM voxel is placed within the MRSI slice, the data from the additional tumor patient measured were used. In this patient, it was ensured that the STEAM voxel was placed completely within the MRSI slice. The results are presented in the Supporting Information.

### 2.4 Statistics

Because metabolite concentrations show regional variations in the brain, four VOIs were defined in order to compare the methods in the healthy volunteers: “Anterior,” “Posterior,” “Left,” and “Right.” The VOIs were manually defined on the MNI‐152‐brain T_1_‐weighted image in the MNI space.^39^ The VOIs were then transformed into the subject‐native space for each of the 5 volunteers using FSL, and analyses were carried out. In each of these VOIs, the concentrations of total tNAA, tCr, and tCho, obtained using the three methods, were compared. Non‐parametric Friedman's repeated‐measures analysis of variance was used to assess the significance of the difference between the methods, with post hoc Durbin‐Conover tests carried out to determine the groups with significant differences. This was carried out for each of the VOIs and each of the metabolites separately. The adjusted *p*‐value threshold for significance (after Bonferroni correction) was 0.01. Hence, *p* < 0.01 was considered significant. Bland–Altman analysis was performed for the Ref‐method‐with‐qMRI and the Proposed‐method, as well as for the Ref‐method‐with‐qMRI and the Ref‐method.

## 3 RESULTS

**FIGURE 2:**
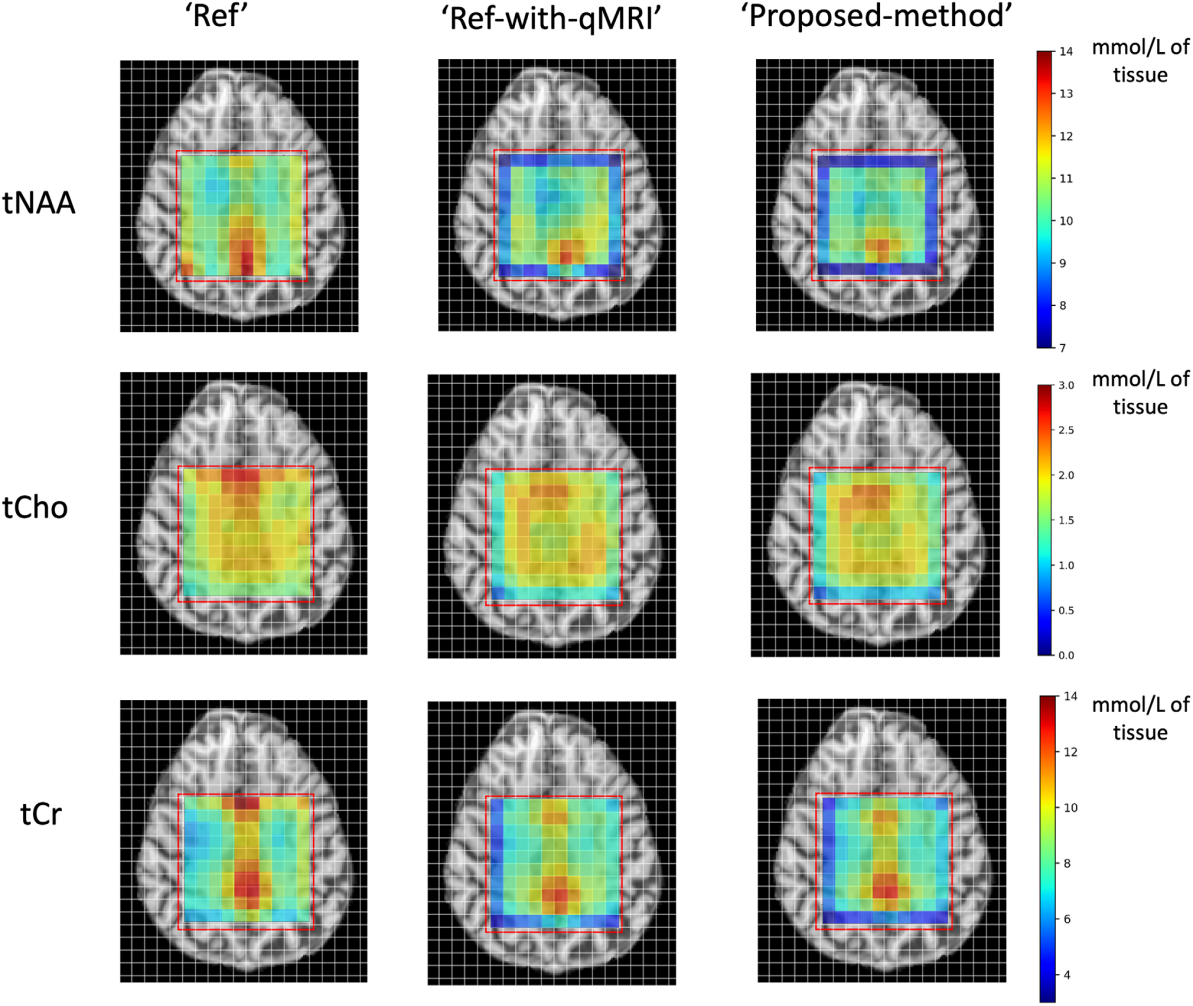
Molar concentration metabolite maps of Subject 2 obtained with the three methods. The Ref‐method‐with‐qMRI and Proposed‐method agree well with each other. The Ref‐method maps qualitatively differ slightly from the Ref‐method‐with‐qMRI maps. The red box denotes the spectroscopic region of interest. tCho, total choline; tCr, total creatine; tNAA, total N‐acetylaspartate.

Figure 2 shows the metabolite maps obtained using the three methods. Following visual inspection of the metabolite maps, the Ref‐method‐with‐qMRI and Proposed‐method maps showed good agreement. The Ref‐method maps on the other hand differed noticeably, with higher metabolite concentrations in the non‐WM regions, in comparison to the Ref‐method‐with‐qMRI and Proposed‐method maps.

**FIGURE 3:**
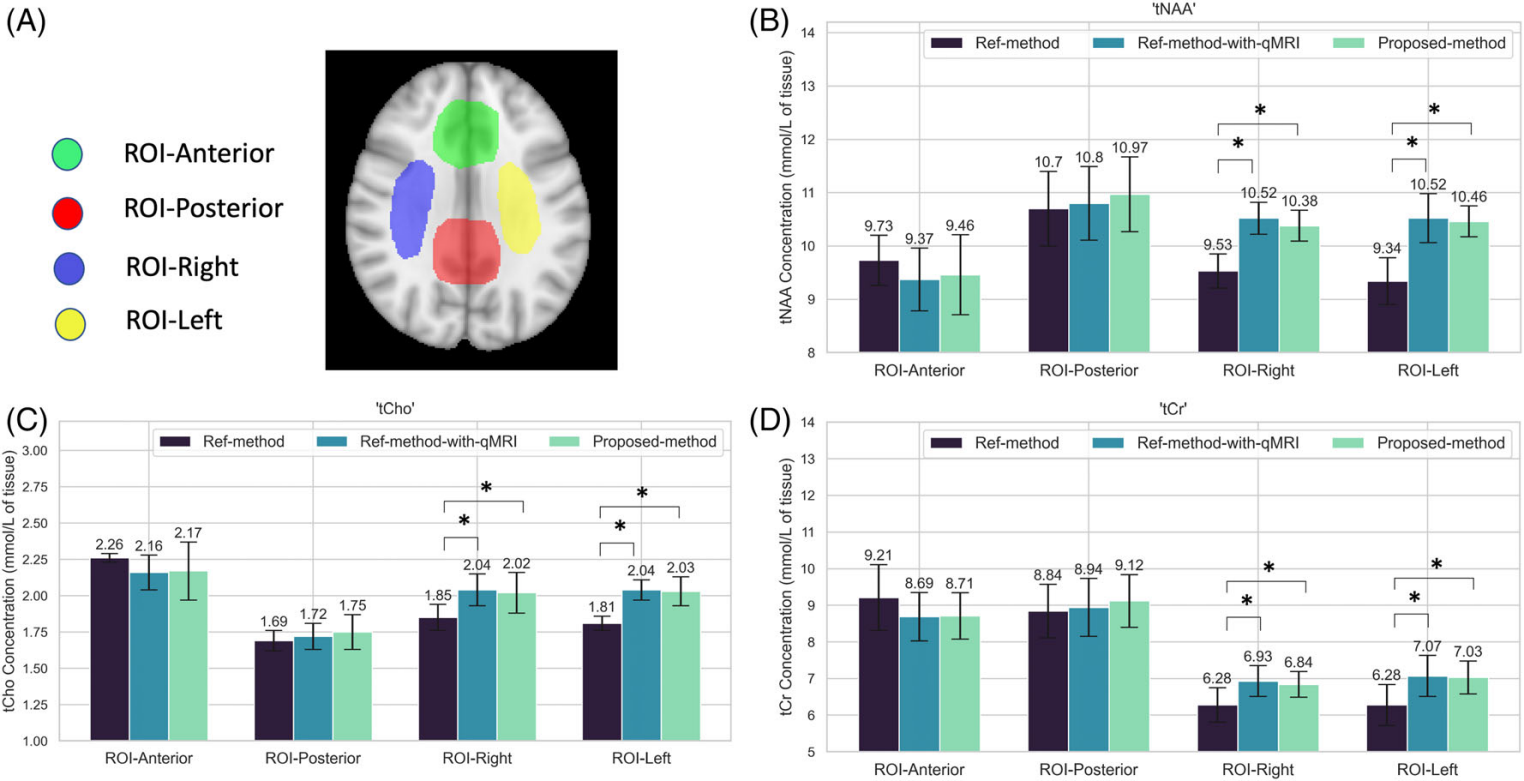
(A) The regions of interest (ROIs) defined on the MNI template. (B–D) Box plots showing the mean metabolite molar concentrations averaged across the 5 subjects for the different ROIs for total N‐acetylaspartate (tNAA), total choline (tCho), and total creatine (tCr), respectively. The error bars denote the standard deviation across the 5 healthy subjects. The stars denote significant differences (*p* < 0.01).

Figure 3 shows bar plots for mean “tNAA,” “tCr,” and “tCho” values across the 5 healthy subjects, as well as the four VOIs, defined in the MNI space (upper left). In the anterior and posterior VOIs, where GM and CSF are predominant, the metabolite values did not differ among the three methods (*p*‐values displayed in Table 3). In the left and right regions of interest (ROIs), however, which predominantly consist of WM, the Ref‐method values were significantly lower than the values obtained using the Ref‐method‐with‐qMRI (Table 3). There were no significant differences, however, between metabolite concentrations obtained with the Proposed‐method and those obtained with the Ref‐method‐with‐qMRI. Metabolite concentrations derived from each of the three methods and the defined ROIs are presented in Table 4.

**TABLE 3:** *p*‐Values for pooled metabolite concentration differences between the Ref‐method, Ref‐method‐with‐qMRI, and proposed method across the 5 healthy subjects.

	tNAA	tCho	tCr
ROI‐Anterior	*p* = 0.549	*p* = 0.449	*p* = 0.449
ROI‐Posterior	*p* = 0.449	*p* = 0.247	*p* = 0.449
ROI‐Left	*p* < 0.01	*p* < 0.01	*p* < 0.01
ROI‐Right	*p* < 0.01	*p* < 0.01	*p* < 0.01

Abbreviations: qMRI, quantitative MRI; ROI, region of interest; tCho, total choline; tCr, total creatine; tNAA, total N‐acetylaspartate.

**TABLE 4:** Intersubject mean (± standard deviation [SD]) molar and molal metabolite concentrations in regions of interest (ROIs) obtained using the three methods. The ± SD represents the standard deviation of the mean ROI metabolite concentrations across the 5 subjects.

		Ref‐method		Ref‐method‐with‐qMRI		Proposed‐method	
		Molar [mmol/L]	Molal [mmol/kg]	Molar [mmol/L]	Molal [mmol/kg]	Molar [mmol/L]	Molal [mmol/kg]
ROI‐Anterior	tNAA	9.73 ± 0.47	12.61 ± 0.43	9.37 ± 0.59	13.14 ± 0.79	9.46 ± 0.75	13.02 ± 0.56
	tCho	2.26 ± 0.03	2.92 ± 0.02	2.15 ± 0.12	3.06 ± 0.17	2.17 ± 0.20	3.02 ± 0.18
	tCr	9.21 ± 0.90	11.90 ± 0.99	8.69 ± 0.66	12.53 ± 1.60	8.71 ± 0.63	12.27 ± 1.02
ROI‐Posterior	tNAA	10.70 ± 0.70	13.64 ± 0.82	10.80 ± 0.69	13.45 ± 1.06	10.97 ± 0.70	13.57 ± 0.65
	tCho	1.69 ± 0.07	2.16 ± 0.07	1.72 ± 0.09	2.13 ± 0.10	1.75 ± 0.12	2.16 ± 0.10
	tCr	8.84 ± 0.73	11.26 ± 0.85	8.94 ± 0.79	11.07 ± 1.04	9.12 ± 0.72	11.22 ± 0.75
ROI‐Right	tNAA	9.53 ± 0.32	13.22 ± 0.40	10.52 ± 0.30	13.90 ± 0.45	10.38 ± 0.29	13.71 ± 0.44
	tCho	1.85 ± 0.09	2.57 ± 0.12	2.04 ± 0.11	2.70 ± 0.14	2.02 ± 0.14	2.67 ± 0.18
	tCr	6.28 ± 0.47	8.71 ± 0.60	6.93 ± 0.42	9.15 ± 0.56	6.84 ± 0.35	9.03 ± 0.46
ROI‐Left	tNAA	9.34 ± 0.44	12.97 ± 0.56	10.52 ± 0.46	13.83 ± 0.60	10.46 ± 0.29	13.75 ± 0.44
	tCho	1.81 ± 0.05	2.51 ± 0.07	2.04 ± 0.07	2.68 ± 0.10	2.03 ± 0.10	2.67 ± 0.15
	tCr	6.28 ± 0.56	8.71 ± 0.72	7.07 ± 0.56	9.28 ± 0.66	7.03 ± 0.45	9.23 ± 0.52

Abbreviations: qMRI, quantitative MRI; tCho, total choline; tCr, total creatine; tNAA, total N‐acetylaspartate.

Voxels located on the outer borders of the ROI in MRSI are commonly deemed unreliable because of the imperfect slice profile and ignored in the analysis. Interestingly, as noticeable in Figure 2, the bordering voxels reveal differences among the three methods, with a notable underestimation of the metabolite values obtained using the Ref‐method‐with‐qMRI and the Proposed‐method, and a slight overestimation of metabolite values in the Ref‐method.

**FIGURE 4:**
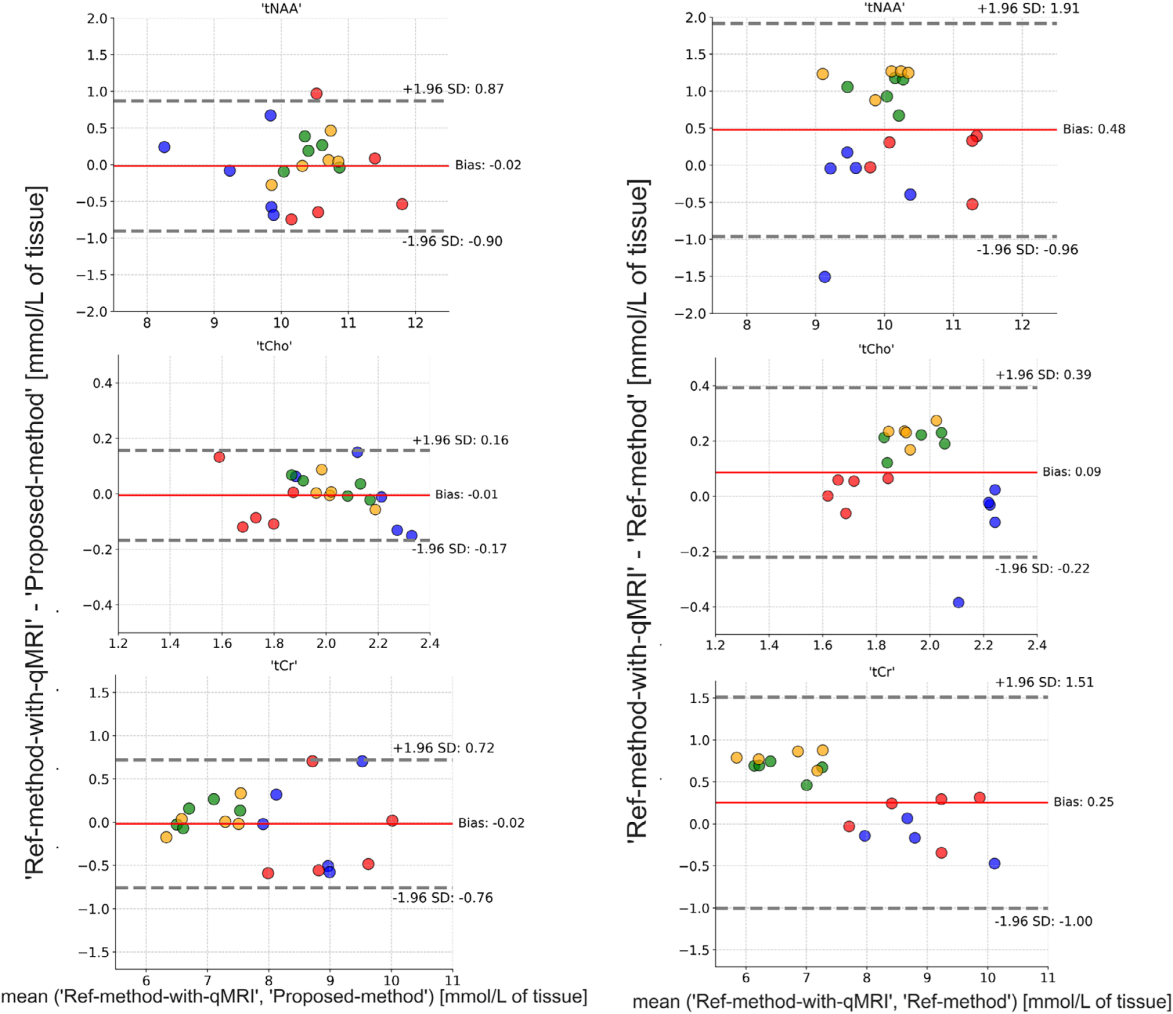
Bland–Altman analyses. *Left column*: Difference between the Ref‐method‐with‐qMRI and the Proposed‐method are plotted on the y‐axis; x‐axis shows the mean of the metabolite concentrations obtained using the two methods. *Right column*: Difference between the Ref‐method‐with‐qMRI and Ref‐method are plotted on the y‐axis; x‐axis shows the mean of the metabolite concentrations obtained using the two methods. Data points by color: green, region of interest (ROI) anterior; red, ROI posterior; blue, ROI right; yellow, ROI left. qMRI, quantitative MRI; SD, standard deviation.

Figure 4 shows the results of the Bland–Altman analysis for the comparison of the Ref‐method‐with‐qMRI and Proposed‐method, as well as the Ref‐method‐with‐qMRI and Ref‐method.

Regarding interchangeability of the methods, the Ref‐method‐with‐qMRI and Proposed‐method were in agreement with a small bias of less than 0.5% and a standard deviation (SD) of less than 10% of the mean metabolite concentrations. The differences between Ref‐method‐with‐qMRI and Ref‐method showed a larger bias (˜5%) and SD (˜20%) in comparison to the differences between Ref‐method‐with‐qMRI and Proposed‐method.

**FIGURE 5:**
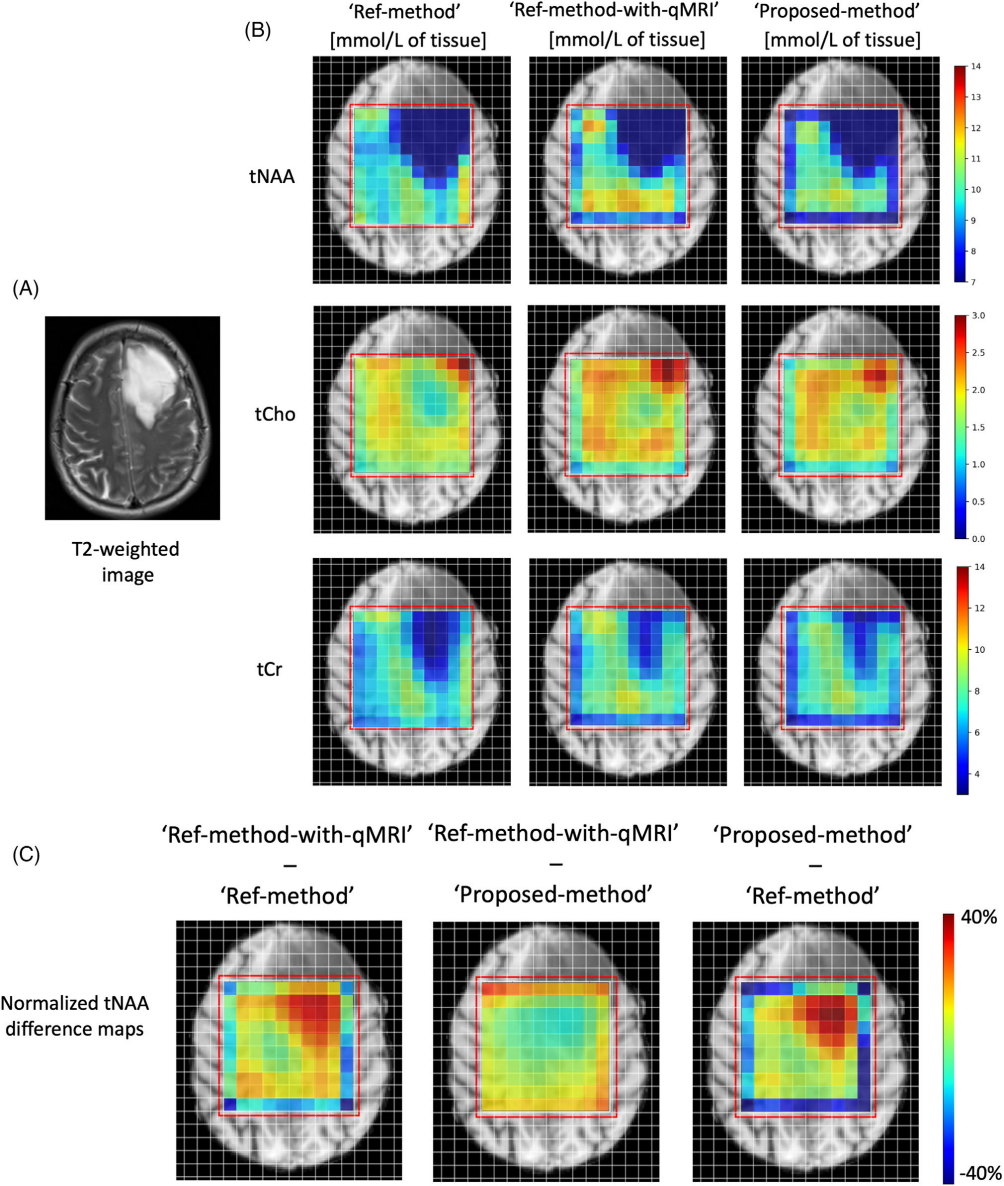
Metabolite concentration maps in a brain tumor (BT) patient (diagnosis: astrocytoma, IDH‐mutant, WHO grade 3). (A) T_2_‐weighted image showing an intra‐axial BT in the left frontal lobe. (B) The metabolite maps obtained using Ref‐method, Ref‐method‐with‐qMRI, and Proposed‐method. As expected, all methods show a decrease in total N‐acetylaspartate (tNAA) and total creatine (tCr), and an increase in tCho concentrations in the tumor area. (C) Difference maps of the tNAA are shown. The “Ref‐method‐with‐qMRI – Ref method” and “Ref‐method‐with‐qMRI – Proposed‐method' difference maps were normalized to the “Ref‐method‐with‐qMRI” tNAA map. The “Proposed‐method – Ref‐method” difference map was normalized to the Proposed‐method tNAA map. In the tumor region, there is an underestimation of the metabolite values obtained using the Ref‐method.

Figure 5 shows the results of application of the three quantification methods in a BT patient. Qualitatively, the maps reveal differences between the Ref‐method and the Ref‐method‐with‐qMRI/Proposed‐method. Difference maps were calculated for quantitative display. The difference maps reveal an overestimation of the metabolite concentrations in the healthy tissue and underestimation of the metabolite concentrations in the tumor tissue with the Ref‐method in comparison to the Proposed‐method and the Ref‐method‐with‐qMRI. In the tumor region, the Ref‐method metabolite concentrations are about 35% lower than those obtained using the other two methods.

As shown in Figure S1, the concentrations measured with the MRSI‐STEAM water reference are 5% to 10% more than that measured using the MRSI‐sLASER water reference. The normalized difference between the two water reference acquisitions is also shown in Figure S1.

## 4 DISCUSSION

In this work, we propose a fast, accurate, and easily implementable qMRI‐based approach for absolute quantification of cerebral metabolites measured with a MRSI sequence. The required mGRE, echo‐planar imaging, and SV‐STEAM sequences are sequences offered by all vendors.

In comparison to other efforts using qMRI for individual‐specific metabolite quantification, our approach is faster for two reasons:

1. A fast protocol for qMRI is used to correct for water relaxation times; and
2. The measurement of the 2D unsuppressed water reference (˜8 min) is avoided, replacing it with the H_2_O map obtained from the qMRI protocol and calibrating it using a fast SV‐STEAM sequence (10 s).

Measuring qMRI enables a more accurate correction of water relaxation, obtaining the true water content of the tissue for calculation of metabolite concentration, and for replacement of a long redundant measurement of 2D unsuppressed water reference (˜8 min) with a fast SV‐STEAM sequence (10 s). The replacement of the unsuppressed water spectrum with the H_2_O map obtained from qMRI is novel in the current work. The Proposed‐method does not rely on T_2_ mapping, as only the T_2_ value in the STEAM voxel (preferably placed in NAWM) needs to be known. The T_2_ value of the STEAM voxel is estimated from the T_2_* value obtained with the qMRI protocol. We hypothesized that this estimation is sufficient for the purpose of metabolite quantification. Our hypothesis was confirmed by the excellent agreement between the Proposed‐method and the Ref‐method‐with‐qMRI, which are based on an additional full T_2_ mapping.

For the proposed method, because the sLASER sequence uses adiabatic pulses, the metabolite signal was relatively insensitive to transmit profile (B_1_^+^) inhomogeneities.^36^ For the STEAM measurement, the B_1_^+^ value was obtained from qMRI. In sequences that do not rely on nonadiabatic pulses (e.g., a PRESS sequence) being used, a correction for B_1_^+^ inhomogeneities for the metabolite signal should be carried out. This correction is readily available from the qMRI protocol used. sLASER was chosen in this study, as it has been shown that sLASER leads to a significant reduction of chemical‐shift displacement artifacts in comparison to PRESS and STEAM.^36^ For spectroscopic data, an in‐line correction option provided by the vendor (“Prescan normalize”) was used to correct the receive field inhomogeneity. Thus, the spectroscopic data (including the unsuppressed water spectrum) obtained from the scanner were already corrected for receive field inhomogeneity. However, in the proposed method, the metabolite MRSI was corrected for the receive field inhomogeneity using “Prescan normalize,” whereas the generation of H_2_O maps relies on a postprocessing bias field correction strategy.^40^ In this work, a good agreement between the ref‐method‐with‐qMRI and the proposed‐method demonstrates that the bias field correction algorithm's performance is similar to that of the vendor‐provided receive field correction strategy. Hence, if a vendor‐based normalization option is not available, this correction can be performed offline. Ideally, the bias field maps generated as part of the postprocessing step in water content mapping could be used, as described by Lecocq et al.^19^

“Prescan normalize” corrects well for the receiver field inhomogeneity within the MRSI slice. This was the case for the healthy volunteers, in whom the STEAM voxel was overlapping/very close to the MRSI acquisition slice. However, when the STEAM voxel is placed away from the MRSI slice in the through‐slice direction, we noticed that an additional correction for the residual receiver field inhomogeneity is required and had to be applied for the BT patient measured in this study. For future studies, we recommend placing the STEAM voxel within the MRSI slice.

Using the Ref‐method led to an underestimation of metabolite concentrations in ROIs with predominantly WM (ROI‐Left and ROI‐left) in the 5 healthy subjects, in comparison to the Ref‐method‐with‐qMRI. ROIs containing predominantly GM and CSF (ROI‐Anterior and ROI‐Posterior) showed no significant difference in the metabolite concentrations obtained using the three methods. This might be a result of the increased uncertainty of the literature and measured qMRI values in CSF voxels or voxels affected by CSF partial volume effects. Even in GM, literature T_2_ values range from 55 ms to 110 ms, making the metabolite concentrations obtained using the Ref‐method highly sensitive to the choice of the GM T_2_ value. The GM T_1_ and water‐content values also have a higher SD across subjects and a broader within‐subject distribution within in comparison to WM. These uncertainties in the GM/CSF‐predominant ROIs lead to an increased SD and a decreased statistical power to detect biases. In WM, on the other hand, qMRI is relatively well‐behaved and shows significantly lower SDs. The literature values of T_1_, T_2_, and H_2_O also fall in a narrower range. The lower error of qMRI mapping and correction can lead to an increased statistical power in the ROIs with predominantly WM. This is also evident in the lower intersubject SD of the metabolite values in ROI‐Left and ROI‐Right as compared with ROI‐Anterior and ROI‐Posterior (Figure 3 and Table 3). Variable flip angle–based T_1_ mapping and T_2_ mapping are known to be less accurate in CSF. In voxels affected by CSF partial volume, the Proposed‐method has the advantage of not using the T_1_ maps directly, but instead the final water content map, which is corrected for T_1_ and T_2_* relaxation in the WM and GM. A constant T_1_ value of 4300 ms is used for correction of the T_1_ relaxation of the CSF voxels; furthermore, in the water content maps, CSF voxels are normalized to a water content value of 1. In summary, the Proposed‐method is less sensitive to inaccuracies of CSF T_1_/T_2_ mapping.

The metabolite concentrations obtained in the healthy volunteers are similar to values reported by others.^9^, ^28^, ^41^, ^42^, ^43^ The discrepancy between the Ref‐method and Ref‐method‐with‐qMRI metabolite values highlights the bias resulting from using literature T_1_ and T_2_ values for quantification and further underlines the importance of actual relaxation‐time measurements, even in healthy subjects. In healthy subjects, regional variations in the T_1_, T_2_, and H_2_O are well known.^44^ Using global T_1_, T_2_, and H_2_O literature values leads to a bias that is confirmed in our study. Even at the relatively short TE of 40 ms used in this study, differences in the metabolite concentrations are observed when relaxation correction was carried out using qMRI. At longer TEs, these differences are only expected to increase, and the correction for water‐relaxation time using measured relaxation parameters will be more crucial in order to obtain accurate metabolite concentrations. A comparison of the metabolite concentrations obtained using MRSI STEAM and that obtained using MRSI sLASER revealed that even after correction of the STEAM half signal, in some regions, the measured STEAM water is 5%–10% lower than the measured sLASER water. This is most likely due to the prolongation of T_2_ relaxation times when adiabatic refocusing pulses are used, as in sLASER.^45^ Acquiring the single‐voxel STEAM with a TE of 20 ms instead of 40 ms reduces this discrepancy between the STEAM and sLASER relaxation times. However, it is important to note that, in all three methods of quantification in this work and also in other similar works in literature, the water acquired using MRS is corrected for water relaxation using T_1_ and T_2_ relaxation times estimated using qMRI.^18^, ^19^, ^20^ In the proposed method, qMRI T_1_ and T_2_ values are used to correct for the water relaxation effects in STEAM, and in the ref‐method‐with‐qMRI, they are used to correct for the water relaxation effects in sLASER. A comparison of qMRI derived water T_1_ and T_2_ values using state‐of‐the‐art methods with MRS‐derived water T_1_ and T_2_ values is warranted in a future study.

In BT patients, segmentation maps usually classify the tumor regions as GM voxels due to increased water content in tumor regions. Using normal GM literature values for water‐relaxation correction in BT regions naturally leads to an underestimation of the local metabolites, which is depicted in the difference maps in Figure 5. Because BT can be highly heterogeneous, it would be impossible to accurately correct for water‐relaxation effects without using qMRI sequences. In the isocitrate dehydrogenase‐mutant astroctyoma patient scanned in this study, the BT regions revealed an approximate 35% difference between the metabolite values obtained using the Ref‐method and the Proposed‐method. Using the Proposed‐method enables patient‐specific quantification of T_1_ and T_2_* relaxation times, which can be used to obtain an accurate relaxation‐corrected spectroscopic water reference, without the need of any literature‐based water relaxation values. Because T_1_ and T_2_* vary widely according to the BT microstructure, type, and size, this patient‐specific measurement becomes even more crucial in accurate absolute quantification of metabolites.

**FIGURE 6:**
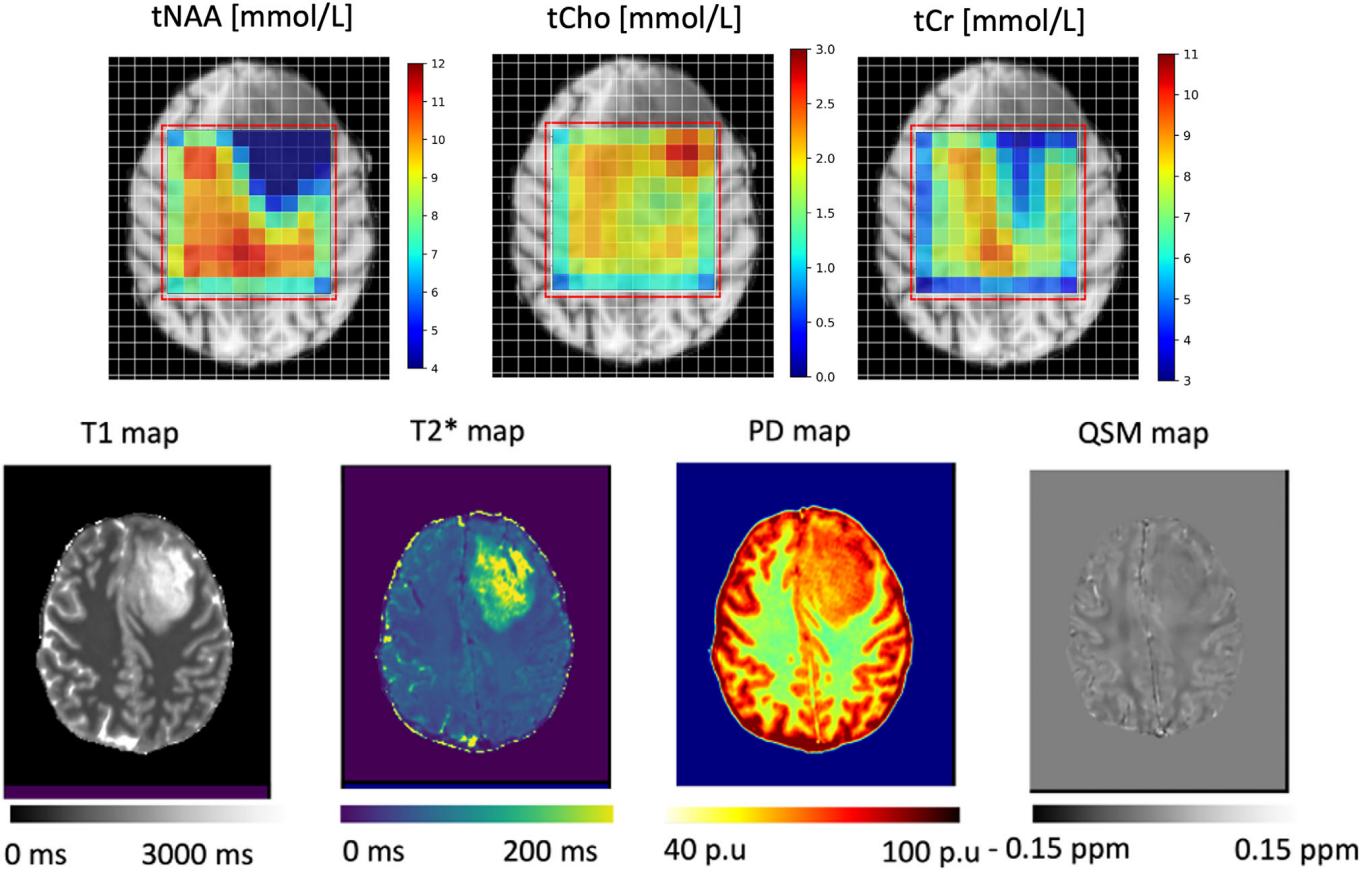
Final outputs of the absolute quantification approach using the Proposed‐method. *Upper row*: Metabolite concentration maps with individual‐specific correction for water‐relaxation effects (“tNAA,” “tCho,” and “tCr” metabolite maps are shown). *Bottom row*: The four quantitative MRI (qMRI) maps (T_1_, T_2_*, H_2_O, and quantitative susceptibility mapping [QSM]) obtained with the qMRI protocol are displayed. PD, proton density; tCho, total choline; tCr, total creatine; tNAA, total N‐acetylaspartate.

To summarize, as demonstrated in Figure 6, the advantage of the proposed method is that four high‐resolution qMRI maps are obtained apart from the absolute metabolite concentrations, without the need of an additional spectroscopic water reference.^46^, ^47^, ^48^ In BT, measuring the actual relaxation times is a necessity for absolute metabolite quantification due to the heterogenous increase in T_1_, T_2_, and water content values,^49^ depending on the tumor type and morphology.

### 4.1 Limitations

One caveat in the proposed method is the underestimation of the water content in large BT.^50^ It has been shown that water content mapping techniques underestimate the water content in large BT, because the bias field correction algorithms mistakenly attribute the increased intensity in BT regions to that arising due to receive field inhomogeneities. In the current study, this was overcome by estimating H_2_O using the T_1_ map for the BT patient data. Future studies are needed to develop novel strategies for accurate correction of the receive field inhomogeneities in large BT. A second limitation of the study is that the metabolite relaxation was corrected for using the relaxation times derived from literature. Ideally, a complete absolute quantification protocol should also include metabolite relaxation time measurements too. However, this is a more complex problem, leading to very long acquisition times for the quantification protocol. Furthermore, for the healthy volunteers, tissue compartment–specific metabolite relaxation times were not used for quantification; instead, average relaxation times from literature were used. A third limitation of the proposed method is that although it enables an accurate quantification of metabolites, it does require an additional time for water quantification, which is close to the acquisition time of the water‐suppressed data. Ideally, for pure MRS studies, qMRI measurements at this high resolution would be deemed unnecessary, as the spectroscopic voxels are of a much lower resolution. However, accurate water content mapping requires data in high resolution, as the calibration with the CSF voxels is a crucial step, and low‐resolution images would lead to increased partial volume of CSF voxels and decreased number of CSF voxels in general. Further studies are required to determine the lower limit of the achievable resolution of water content maps without any bias. A further limitation in this study is the use of the factor “K” for the patient data set, which arises because of the STEAM voxel not being placed in the MRSI field of view. This is confirmed by the results obtained with the additional BT data set (shown in the Supporting Information), which demonstrate that no correction factor is needed if the STEAM voxel is placed within the MRSI field of view. To avoid the differences in “Prescan normalize” correction between the MRSI data and the STEAM voxel, we recommend positioning the STEAM voxel within the MRSI slice in the NAWM. The proposed method further has the limitation that the eddy current correction cannot be performed on a voxel‐wise basis, as the voxel‐wise water spectrum is not acquired. Hence, in approaches where rapidly switching gradients are used, a cautious use of the Proposed‐method is advised.

## Supporting information


**Data S1.** Supporting information.
