# Supplementary material for: Absolute quantification of cerebral metabolites using two‐dimensional proton MR spectroscopic imaging with quantitative MRI‐based water reference
Source: Magn Reson Med. 2025 Aug 13;95(1):4–19. doi: 10.1002/mrm.70027 (PMC12620158; doi:10.1002/mrm.70027)
Supplement: Supplementary file 1 — Data S1. Supporting information. [file MRM-95-4-s001.docx]

**Supporting Information**

**Theory (Ref-method and Ref-method-with-qMRI)**

*Reference method (“Ref-method”)*

In this approach, the unsuppressed water signal (S*_w_*) is obtained using the same MRSI sequence with the same parameters but without water suppression. It is therefore proportional to the number of moles of water in each voxel. By using the same sequence to acquire both the metabolites and water signals, the effect of the transmit and receive field inhomogeneities and other scaling factors are expected to be identical. However, there are two important corrections that need to be carried out for absolute quantification: 1) T1 and T2 relaxation effects of water and metabolites and 2) correction for CSF partial-volume effects. The need for the first correction arises from the fact that in order to acquire data in reasonable acquisition times, a short TR (~2s) is often used, and hence the signal is never fully relaxed. Further, hardware constraints and the sLASER sequence with 4 adiabatic full passage pulses limit the minimum achievable echo time, which leads to a non-zero T2 relaxation of the signal. The need for the second correction for CSF partial volume effects arises from the fact that the metabolites concentrations in CSF are usually negligible, hence, only the concentration of the metabolites in the non-CSF water are of interest. Including these corrections in the quantification gives us:

$\left[ M \right]_{\mathrm{molal}}(ref)=\frac{S_{M} \cdot R_{W}}{S_{W} \cdot R_{M} \cdot\left( 1-f_{\mathrm{CSF}_{H2O}} \right)}{\cdot[H_{2}O]}_{\mathrm{molal}}$ ……………. (3)

where, $\left[ M \right]_{\mathrm{molal}}\left( ref \right)$ refers to the metabolite concentration obtained using the reference method. $R_{M}$ and $R_{W}$refer to the relaxation correction factors for the metabolites and water relaxation respectively. T1_w_ and T2_w_ refer to the water T1 and T2 relaxation times, and T1_M_ and T2_M_ refer to the metabolite T1 and T2 relaxation times, which are obtained from literature for gray matter (GM) and white matter (WM) regions^29–33^. The molality of water (${[H_{2}O]}_{\mathrm{molal}}$) is 55100 mmol/kg. $f_{\mathrm{CSF}_{H2O}}$ refers to the CSF molal water fraction, as described by Gasparovic et. al^34^. In order to calculate $f_{\mathrm{CSF}_{H2O}}$, T1-weighted images are used to first compute WM and GM segmentations using SPM^35^. The segmentations provide f_WM_, f_GM_ and f_CSF_ which represent the volume fractions of WM, GM and CSF respectively. As described by Gasparovic et. al^34^. $f_{\mathrm{CSF}_{H2O}}$ can then be obtained as follows:

$f_{\mathrm{CSF}_{H2O}}=\frac{f_{CSF}\cdot d_{CSF}}{f_{WM}\cdot d_{WM}+f_{GM}\cdot d_{GM}+f_{CSF}\cdot d_{CSF}}$ ….…………. (4)

where, $d_{\mathrm{WM}}$, $d_{\mathrm{GM}}$ and $d_{\mathrm{CSF}}$ represent the relative molal water density in the WM, GM and CSF respectively, obtained from literature. $d_{\mathrm{WM}}$, $d_{\mathrm{GM}}$ and $d_{\mathrm{CSF}}$ were set to fixed values of 0.65, 0.78 and 0.97 in the original equation described by Gasparovic et. al, but here, these have been replaced by the respective notations and the values used have been obtained from publications which use the current state-of-the-art water content mapping methods (Supporting Information Table S1). Note that f_WM_, f_GM_ and f_CSF_ here represent the segmentations corrected for the difference in the point-spread function (PSF) between the high-resolution imaging data and the low-resolution MRSI data, as detailed in the Supporting Information. $R_{M}$ can be computed as follows:

$R_{M}= e^{\frac{-TE}{T_{2_{M}}}} \cdot(1-e^{\frac{-TR}{T_{1_{M}}}})$ ..….…………. (5)

To compute $R_{W}$, the relaxation time correction factors for each of the different tissue compartments need to be computed separately, i.e,

$$R_{w_{WM}}= e^{\frac{-TE}{T_{2_{w_{WM}}}}} \cdot\left( 1-e^{\frac{-TR}{T_{1_{w_{WM}}}}} \right);R_{w_{GM}}=e^{\frac{-TE}{T_{2_{w_{GM}}}}} \cdot\left( 1-e^{\frac{-TR}{T_{1_{w_{GM}}}}} \right);$$

$R_{w_{CSF}}=e^{\frac{-TE}{T_{2_{w_{GM}}}}} \cdot(1-e^{\frac{-TR}{T_{1_{w_{GM}}}}})$ …….. (6)

where,$R_{w_{x}}$ is the relaxation times correction factor for the tissue segmentation class *x*. $R_{W}$ can be then computed the sum of the product of the individual tissue segmentation class molal water fraction and the corresponding relaxation time correction factors:

$R_{W}=f_{\mathrm{WM}_{H2O}}\cdot R_{w_{WM}}+f_{\mathrm{GM}_{H2O}}\cdot R_{w_{GM}}+f_{\mathrm{CSF}_{H2O}}\cdot R_{w_{CSF}}$  ..…. (7)

In order to compute $\left[ M \right]_{\mathrm{molar}}(ref)$, one can derive the equation similar to equation 5, but by using the tissue molar volume fractions instead of the tissue molal water fractions and replacing ${[H_{2}O]}_{\mathrm{molal}}$with ${[H_{2}O]}_{\mathrm{molar}}$ (Molarity of pure water), as has been well described in Ref^36,37^. Here, however, we compute the metabolite molar concentration ($\left[ M \right]_{\mathrm{molar}}(ref)$), (excluding CSF) directly from $\left[ M \right]_{\mathrm{molal}}(ref)$, by multiplying $\left[ M \right]_{\mathrm{molal}}(ref)$ by the water density in the voxel (excluding CSF):

$\left[ M \right]_{\mathrm{molar}}(ref)=\left[ M \right]_{\mathrm{molal}}(ref)\cdot\frac{f_{WM}\cdot d_{WM}+f_{GM}\cdot d_{GM}}{f_{WM}+f_{GM}}\cdot\rho_{w}$ …. (8)

Here, $\rho_{w}$ represents the density of pure water [kg/L] and was assumed to be equal to unity. Equation 8 gives a direct relation between the molal concentrations (corrected for CSF partial volume) and the molar concentrations (corrected for CSF partial volume) when WM and GM segmentations are available.

*Reference method with qMRI (**“Ref-method-with-qMRI”)*

In the ‘Ref-method’, parameters related to the water obtained from literature are: $T_{2_{WM}}$, $T_{2_{GM}}$, $T_{2_{CSF}}$, $T_{1_{WM}}$, $T_{1_{GM}}$, $T_{1_{CSF}}$ $d_{WM}$ and $d_{GM}$. In the ‘Ref-method-with-qMRI’ , these parameters are measured using qMRI sequences for T2 mapping, T1 mapping, and H_2_O mapping relying on vendor-based sequences^29^. The parameters of acquisition for the qMRI sequences are described in the methods section. In equation 4, for the Ref-method-with-qMRI, the computation of $f_{\mathrm{CSF}_{H2O}}$ and $R_{w}\left( T1,T2 \right)$ changes. In this case, the molal water fraction of the CSF obtained using ‘Ref-method-with-qMRI’ ($f_{\mathrm{CSF}_{{H20}_{qMRI}}}$) is computed using the water content map (${H_{2}O}_{map}$):

$f_{\mathrm{CSF}_{{H20}_{qMRI}}}=\frac{f_{CSF}}{{H_{2}O}_{map}}$ .…. (9)

$R_{w}$ obtained using ‘Ref-method-with-qMRI’ ($R_{W_{qMRI}}$), is computed using the T1 and T2 maps ($T_{1_{map}}$ and $T_{2_{map}}$), as follows:

$R_{W_{qMRI}}\left( T1,T2 \right)=\frac{e^{\frac{TE}{T_{2_{map}}}}}{1-e^{\frac{-TR}{T_{1_{map}}}}}$ …. (10)

The final metabolite concentrations are then calculated according to the following equations (similar to equations 5 and 10):

$\left[ M \right]_{\mathrm{molal}}(ref\_qmri)=\frac{S_{M}\cdot R_{M}}{S_{w}\cdot R_{w_{qMRI}} \cdot\left( 1-{CSF}_{w_{qMRI}} \right)}{\cdot[H_{2}O]}_{\mathrm{molal}}$ ……. (11)

$\left[ M \right]_{\mathrm{molar}}(ref\_qmri)=\left[ M \right]_{\mathrm{molal}}(ref\_qmri)\cdot\frac{\left( {H_{2}O}_{map}-f_{CSF} \right)}{\left( 1-f_{CSF} \right)}\cdot\rho_{w}$ …. (12)

Note that the ${H_{2}O}_{map}$,$T_{1_{map}}$ and $T_{2_{map}}$ are corrected for the difference in PSF due to the differing resolution between MRSI and imaging as detailed in the Supporting Information.

**Supporting Information Table S1**: Literature relaxation times and relative proton densities of water used

|  | WM | GM | CSF |
| --- | --- | --- | --- |
| T1 | 878 ms ^29^ | 1425 ms ^29^ | 4300 ms ^30^ |
| T2 | 58.68 ms ^31^ | 69.45 ms ^31^ | 2000 ms ^32^ |
| H_2_O | 69.99 p.u^29^ | 81.72 p.u^29^ | 100.00 p.u |

**Supporting Information Table S2**: Literature relaxation times of metabolites used ^33^

|  | tNAA | tCr | tCho |
| --- | --- | --- | --- |
| T1 | 1410 ms | 1350 ms | 1190 ms |
| T2 | 271 ms | 154 ms | 197ms |


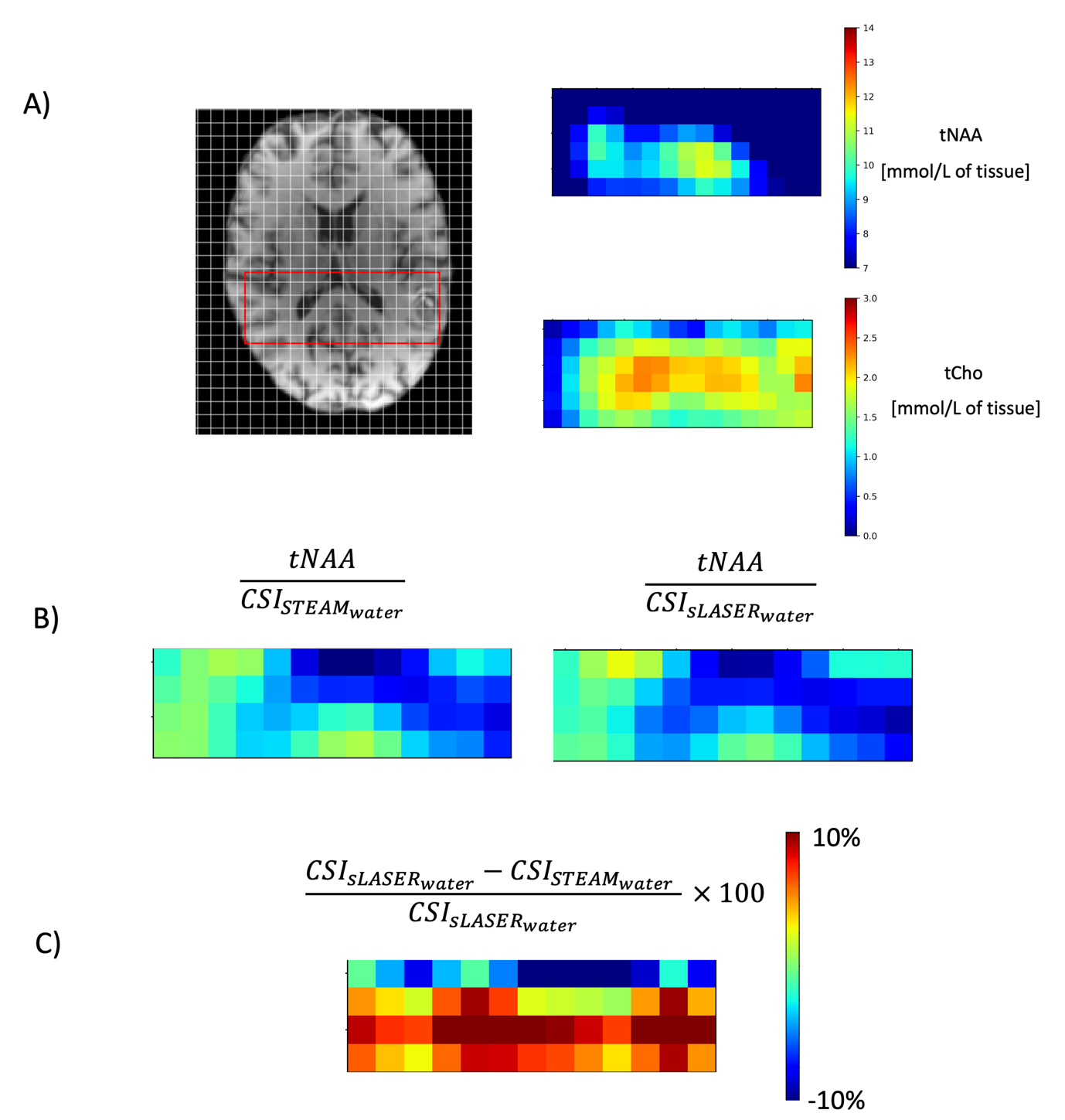


**Figure S1:** **A):** VOI positioning in the brain tumor patient is overlaid on a T1-weighted anatomical image. tNAA and tCho maps are shown as well. Quantitative tNAA and tCho maps obtained using the proposed-method are shown as well. **B):** The tNAA metabolite signal normalized with the CSI_STEAM water reference is shown on the left, and the same metabolite signal normalized to the CSI sLASER water reference is shown on the right. The concentrations are uncorrected for metabolite and water relaxation time effects. **C):** The voxel-wise difference between the sLASER water ref and the STEAM water ref normalized to the sLASER water ref is shown as a percentage. It is seen that the sLASER water ref has a maximum of 10 percent higher signal as compared to the STEAM water ref.

**PSF correction of qMRI maps and WM, GM and CSF segmentations**

The correction of the difference in the point spread functions between the low-resolution 2D MRSI spectroscopic data and the high-resolution imaging data is described in this section. The correction is specific for Siemens VE11c software version and will be different for data acquired with MRI scanners from other vendors. For the current study, the algorithm for PSF is as follows:

Let the ‘*HR_map’* denote the high-resolution qMRI map/ segmentation. And let ‘*HR_map_resliced’* denote the HR_map resliced into the spectroscopic space.

import numpy as np

matrix_size = [X, Y] # Spectroscopic Matrix

middle_point = [X/2, Y/2] # Middle point of the matrix

hamm_width = Width of the hamming filter used # default in Siemens = 0.5

data_PSF = zeros(HR_map_resliced.shape) # Initialize PSF corrected HR image

for i in range(no.of slices (HR_map_resliced*)*):

    data_i = HR_map_resliced [:,:,i]

    data_i(voxels outside VOI) = 0 # Set pixels outside the spectroscopic VOI to 0

    inversefft = np.fft.ifftshift(np.fft.ifft2(data_i)) *# Inverse fft*


    filt = zeros([data_i.shape[0], data_i.shape[1]])

    filt_mod_hamm = zeros(matrix_size)
    ka_image = zeros(matrix_size)

    for x in range(X):
       for y in range(Y):

        k1_max = (x/2 - 0.5)
         k2_max = (y/2 - 0.5)

        delta_k = np.sqrt(((x - middle_point[0])/k1_max)**2 +

((y - middle_point[1])/k2_max)**2)

         ka = (delta_k - (1 - hamm_width))/hamm_width
         ka_image[x,y] = ka
                       
         if ka < 0:
            filt_mod_hamm[x,y] = 1

          else if ka > 1:
             filt_mod_hamm[x,y] = 0.08

          else:
             filt_mod_hamm[x,y] = 0.54 + 0.46*np.cos(np.pi*ka)

          filt[(data_i.shape[0]/2 – X/2): (data_i.shape[0]/2 + X/2),
              (data_i.shape[1]/2 - Y/2): (data_i.shape[1]/2 + Y/2)] = filt_mod_hamm

        inversefft_PSF = inversefft * filt

        data_PSF[:,:,i] = = np.abs(np.fft.fft2(np.fft.fftshift(inversefft_PSF)))

**MRSinMRS checklist**

the Minimum Reporting Standards for in vivo- Magnetic Resonance Spectroscopy (MRSinMRS) checklist can be found in **Table S3**.

**Table S3**. MRSinMRS checklist for our multi-sequence MRS protocol.

| Goethe University Frankfurt |  |  |  |
| --- | --- | --- | --- |
| 1. Hardware |  |  |  |
| a. Field strength [T] | 3 T | 3 T | 3 T |
| b. Manufacturer | Siemens | Siemens | Siemens |
| c. Model (software version if available) | Prisma (VE11C) | Prisma (VE11C) | Prisma (VE11C) |
| d. RF coils: nuclei (transmit/ receive), number of channels, type, body part | 20 ch ^1^H head coil | 20 ch ^1^H head coil | 20 ch ^1^H head coil |
| e. Additional hardware | N/A | N/A | N/A |
| 2. Acquisition |  |  |  |
| a. Pulse sequence | 2D ^1^H Semi-LASER CSI (vendor-provided) | 2D ^1^H Semi-LASER CSI (vendor-provided, water reference) | ^1^H STEAM SVS (vendor-provided, water reference) |
| b. Volume of Interest (VOI) locations | Healthy volunteer: above the corpus callosum  Patient: tumor and contralateral | Healthy volunteer: above the corpus callosum  Patient: tumor and contralateral | Normal-appearing white matter |
| c. Nominal VOI size [cm^3^, mm^3^] | Adjusted according to tumor volume with a slice thickness of 12 mm | Adjusted according to tumor volume with a slice thickness of 12 mm | 10 x 10 x 10 mm^3^ |
| d. Repetition Time (TR), Echo Time (TE) [ms, s] | TR = 2000 ms, TE = 40 ms | TR = 2000 ms, TE = 40 ms | TR = 10000 ms, TE = 20 ms, TM = 10 ms |
| e. Total number of Excitations or acquisitions per spectrum  In time series for kinetic studies   1. Number of Averaged spectra (NA) per time-point 2. Averaging method (e.g. block-wise or moving average) 3. Total number of spectra (acquired / in time-series) | 2 | 1 | 1 |
| f. Additional sequence parameters  (spectral width in Hz, number of spectral points, frequency offsets)  If STEAM:, Mixing Time (TM)  If MRSI: 2D or 3D, FOV in all directions, matrix size, acceleration factors, sampling method | 2000 Hz, 1024 points  delta frequency = −2.7 ppm  flip angle = 90°  2D: 240 × 240 × 12 mm^3^ FOV; matrix size 20 x 20 interpolated to 32 x 32; no acceleration factor; weighted distribution sampling | 2000 Hz, 1024 points  delta frequency = 0 ppm  flip angle = 90°  2D: 240 × 240 × 12 mm^3^ FOV; matrix size 20 x 20 interpolated to 32 x 32; no acceleration factor; | 1200 Hz, 1024 points  delta frequency = 0 ppm  flip angle = 90° |
| g. Water Suppression Method | CHESS | None | None |
| h. Shimming Method, reference peak, and thresholds for “acceptance of shim” chosen | Automated 3D B0 field mapping technique (GRE-SHIM for brain) | Automated 3D B0 field mapping technique (GRE-SHIM for brain) | Automated 3D B0 field mapping technique (GRE-SHIM for brain) |
| i. Triggering or motion correction method  (respiratory, peripheral, cardiac triggering, incl. device used and delays) | N/A | N/A | N/A |
| 3. Data analysis methods and outputs |  |  |  |
| a. Analysis software | LCmodel 6.2 | LCmodel 6.2 | LCmodel 6.2 |
| b. Processing steps deviating from quoted reference or product | Basis set created using jMRUI 6.0 plug-in NMR-ScopeB | LCModel water reference fit | LCModel water reference fit |
| c. Output measure  (e.g. absolute concentration, institutional units, ratio)Processing steps deviating from quoted reference or product | Ratios to water | Used as water reference for metabolite quantification | Used as water reference for metabolite quantification |
| d. Quantification references and assumptions, fitting model assumptions | The basis set included spectra of N-acetylaspartate, N-acetylaspartylglutamate, glycerophosphocholine, choline, creatine, γ-aminobutyric acid, glucose, glutamate, glutamine, myo-inositol, glutathione, glycine, alanine, lactate, valine. | None | None |
| 4. Data Quality |  |  |  |
| a. Reported variables  (SNR, Linewidth (with reference peaks)) | None | None | None |
| b. Data exclusion criteria | LCModel SNR <3, LCModel FWHM >0.1 ppm. | None | None |
| c. Quality measures of postprocessing Model fitting (e.g. CRLB, goodness of fit, SD of residual) | CRLB < 10% for total choline. | None | None |
| d. Sample Spectrum | None | None | None |

LC Model parameters:

$LCMODL

wconc= 1.0

ppmst= 4.0

ppmend= 0.2

nunfil= 1024

ndslic= 1

ndrows= 32

ndcols= 32

ltable= 7

lps= 8

lcsv= 11

key= 210387309

islice= 1

irowst= 1

irowen= 32

icolst= 1

icolen= 32

hzpppm= 1.2326e+02


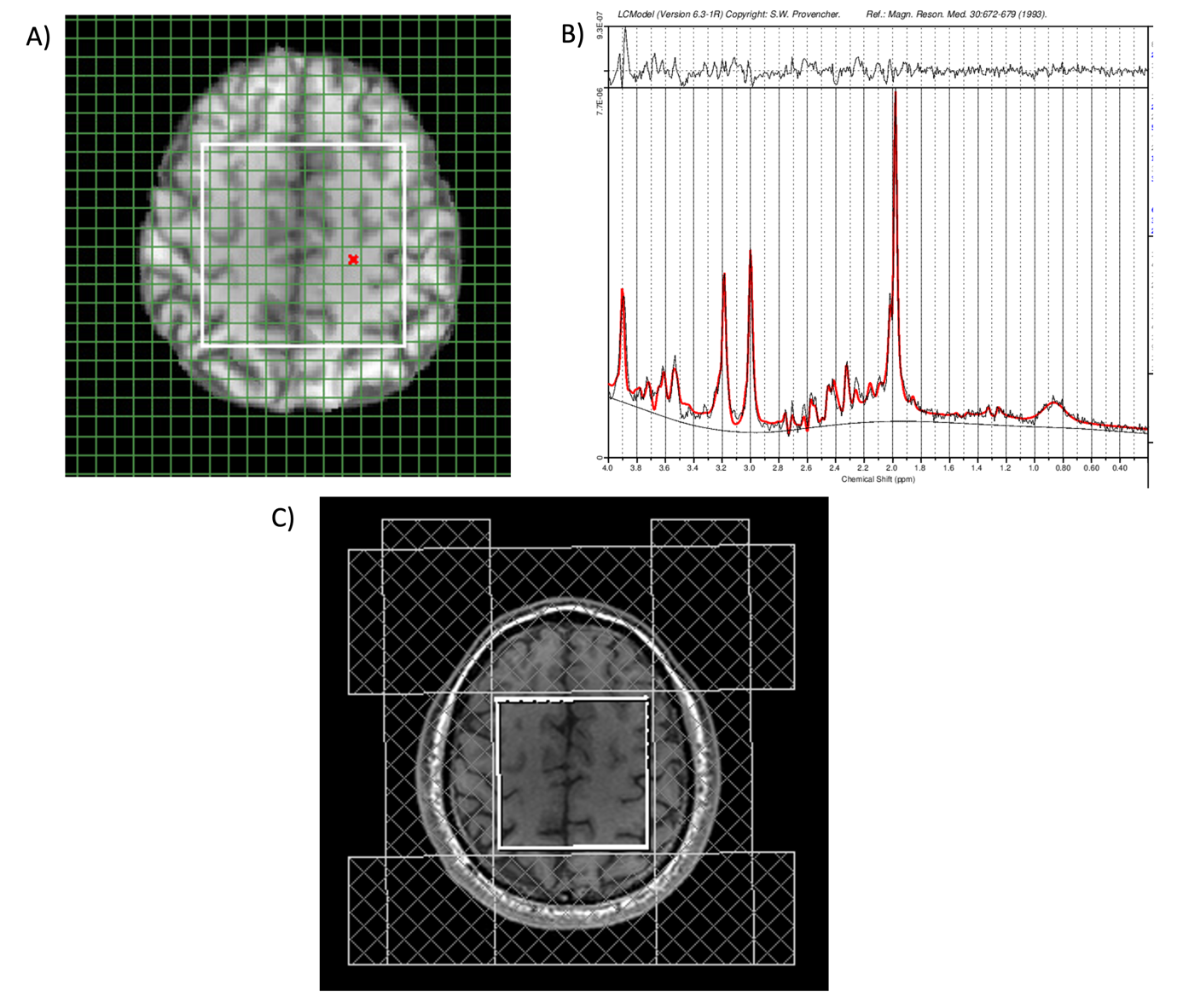


**Figure S2:** **A)** Subject 2 MRSI grid overlaid on the anatomical image. Selected voxel, the spectrum of which is shown in B. **B)** Representative spectrum of the selected voxel. **C)** Planning of the additional saturation bands in Subject 2.

**Additional tumor patient results:**

An additional tumor patient was recruited and scanned with the Proposed method, the Ref-with-qMRI method and the Ref-method. In this patient, it was ensured that the STEAM voxel was placed completely within the slice (**Figure S3**). The tumor location and hence the MRSI slice planning was very similar to that of the patient shown in the manuscript. The correction factor (K) calculated in the same way as was done for the tumor patient data displayed currently in the manuscript, was close to 1. The metabolite maps for the three methods calculated without using any correction factor are shown in **Figure** **S4**.


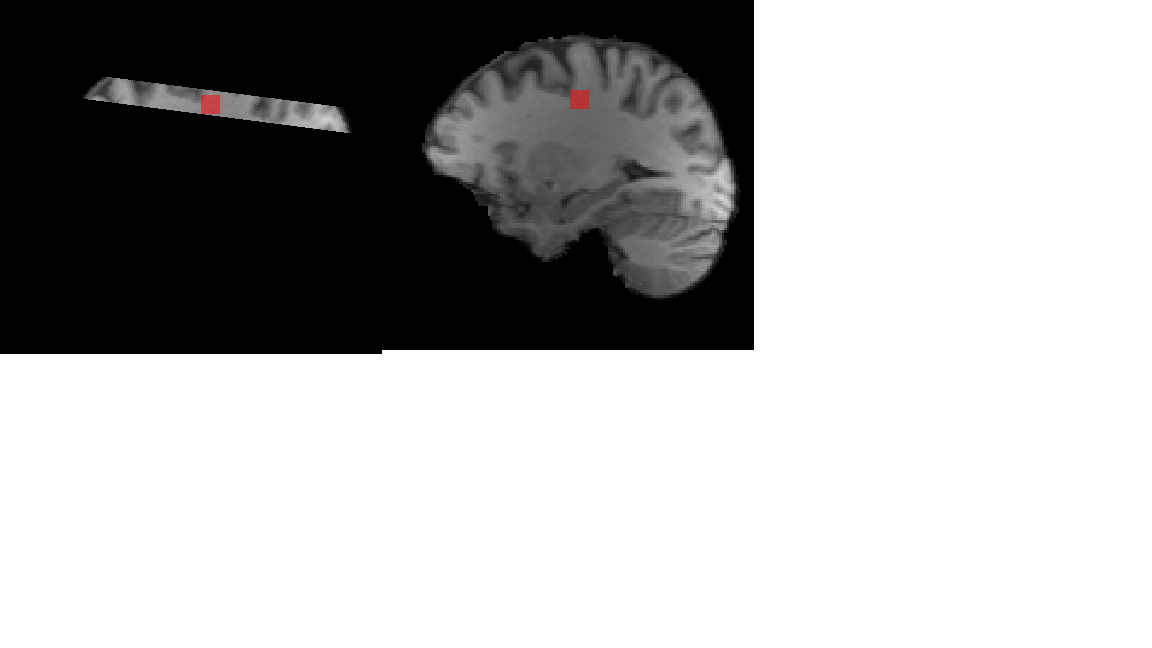


**Figure S3:** STEAM voxel positioning in the patient additionally measured. The STEAM voxel was placed within the MRSI slice.


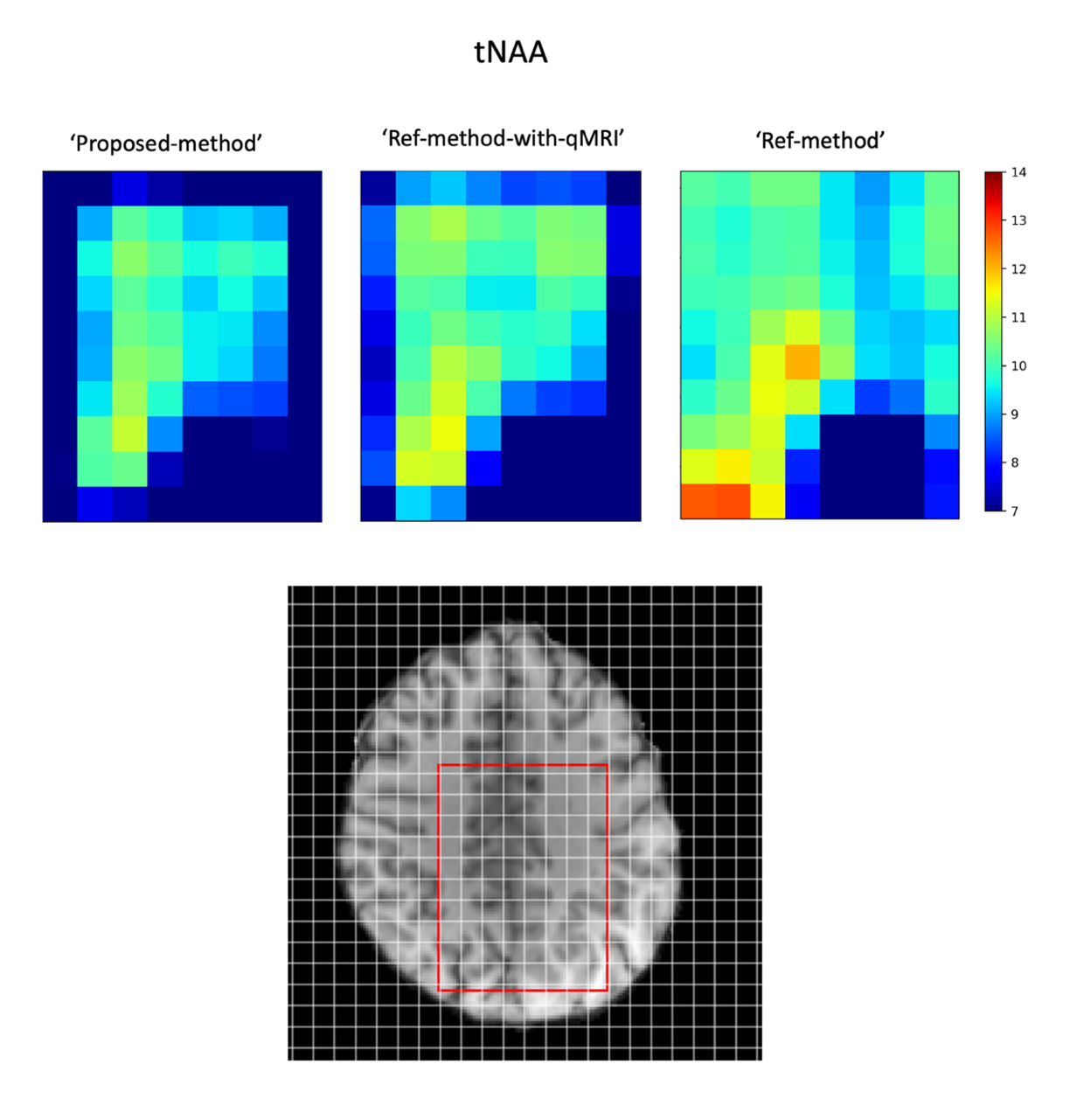


**Figure S4:** tNAA maps obtained in the additional patient measured, without the use of any correction factor K. The “Proposed-method” agrees well with the “Ref-method-with-qMRI”.

The Proposed-method agrees well with the Ref-method-with-qMRI without the need for any correction factor. This further points to the reasoning that the discrepancy seen in the patient currently shown in the manuscript is majorly because of the placement of the STEAM voxel away from MRSI slice, resulting in differences in the “Pre-scan normalize” correction.
